# Differentiation of atypical pancreatic neuroendocrine tumors from pancreatic ductal adenocarcinomas: Using whole‐tumor CT texture analysis as quantitative biomarkers

**DOI:** 10.1002/cam4.1746

**Published:** 2018-08-27

**Authors:** Jiali Li, Jingyu Lu, Ping Liang, Anqin Li, Yao Hu, Yaqi Shen, Daoyu Hu, Zhen Li

**Affiliations:** ^1^ Department of Radiology Tongji Hospital Tongji Medical College Huazhong University of Science and Technology Wuhan China

**Keywords:** atypical pancreatic neuroendocrine, computed tomography, pancreatic ductal adenocarcinomas, texture analysis

## Abstract

**Background:**

To explore the application value of computed tomography (CT) texture analysis in differentiating atypical pancreatic neuroendocrine tumors (pNET) from pancreatic ductal adenocarcinomas (PDAC).

**Materials and methods:**

This single‐center retrospective study was approved by local institutional review board, and the requirement for informed consent was waived. We retrospectively analyzed 127 patients with 50 PDACs and 77 pNETs in pathology database between January 2012 and May 2017.These patients successfully finished preoperative contrast‐enhanced CT test. Texture parameters (mean, median, 5th, 10th, 25th, 75th, 90th percentiles, skewness, kurtosis and entropy) were extracted from portal images and compared between PDAC and 77 pNET groups using proper statistical method. The optimal parameters for differentiating PDACs and atypical pNETs were gained through receiver operating characteristic (ROC) curves.

**Results:**

On the basis of arterial enhancement, 52 pNETs (67%, 52/77) were typical hypervascular and 25 pNETs (32%, 25/77) were atypical hypovascular. Compared with PDACs, atypical pNETs had statistically higher mean, median, 5th, 10th, and 25th percentiles (*P *=* *0.006, 0.024, 0.000, 0.001, 0.021, respectively) and statistically lower skewness (*P *=* *0.017). However, there were no difference for 75th, 90th percentiles, kurtosis and entropy between these two tumors (*P = *0.232, 0.415, 0.143, 0.291, respectively). For differentiating PDACs and atypical pNETs, 5th percentile and 5th+skewness were optimal parameters for alone and combined diagnosis, respectively.

**Conclusion:**

Volumetric CT texture features, especially combined diagnosis of 5th+skewness can be used as a quantitative tool to distinguish atypical pNETs from PDACs.

## 1 INTRODUCTION

Pancreatic neuroendocrine tumors (pNETs) are the second common tumor of pancreas originating from pancreatic islet cells.^1^ Although pNET is characterized by hypervascular on CT arterial phase, recent studies have reported that up to 41.5% of pNETs may show atypical hypovascular enhancement pattern.^2^ These atypical pNETs on CT are difficult to be differentiated from pancreatic ductal adenocarcinomas(PDACs).^3^

Accurate preoperative differentiation between pNET and PDAC is crucial not only for selecting more suitable treatment but patient prognosis.^4^, ^5^ For pNET patients, previous studies have reported that more aggressive surgery method could lead to higher morbidity and did not significantly improve overall survival compared with conservative resections.^6^ While for the treatment of PDACs, more extensive surgical approaches combining with lymph node dissection are commonly suggested.^7^

Contrast‐enhanced CT is currently widely used for evaluating abdominal lesions because of its broad availability and convenience.^8^, ^9^, ^10^ Jung Hoon^11^ and Sun Kyung et al^12^ have evaluated the imaging characteristics of atypical hypovascular PNET and PDAC, respectively. They found duct dilatation in CT may be a helpful predictor for PDAC.^11^ A well‐defined margin and hyper‐ or iso‐enhancement on portal phase may be useful MR imaging features for atypical hypovascular pNETs.^12^ However, the current identification of pancreatic tumors is mostly based on anatomical imaging characteristics, which is still insufficiently specific due to overlapping imaging features and easily affected by subjective of radiologists. Therefore, a method to quantitatively assess differences between tumors is urgently needed.

Texture analysis is an emerging and noninvasive method of assessing organizational characteristics, which can provide objectively quantified parameters for differential diagnosis independent of subjective analysis.^13^ Many prior studies have proved that CT texture analysis (CTTA) can be utilized to characterize a variety of tumors such as gastric cancer, renal neoplasms, and colorectal cancer.^14^, ^15^, ^16^ However, the utility of CT texture analysis to differentiate atypical pNET and PDAC has not been widely reported. Moreover, most of the previous studies chose one aspect of tumor as a region of interest (ROI), which did not reflect the characteristics of the entire tumor and likely to result in wrong results due to selection bias.^17^

Therefore, this study selected the entire tumor as ROI for texture analysis and explored the optimal parameters for identifying PDAC and atypical pNET on contrast‐enhanced CT.

## 2 MATERIALS AND METHODS

### 2.1 Patients

This retrospective study was approved by institutional review board of our hospital, and the requirement for informed consent was waived. This study identified a total of 105 patients with surgery pathologically confirmed pNET through retrospectively analyzing the pathology database between January 2012 and May 2017. Then, the radiological database was searched for further confirming radiographic information. Twenty‐eight patients were excluded based on the following criterion: no enhanced CT images (n = 10); Insufficient image quality (n = 3); lesions invisible on CT images (n = 4); and history of local treatment or systemic chemotherapy (n = 11).

**Figure 1:**
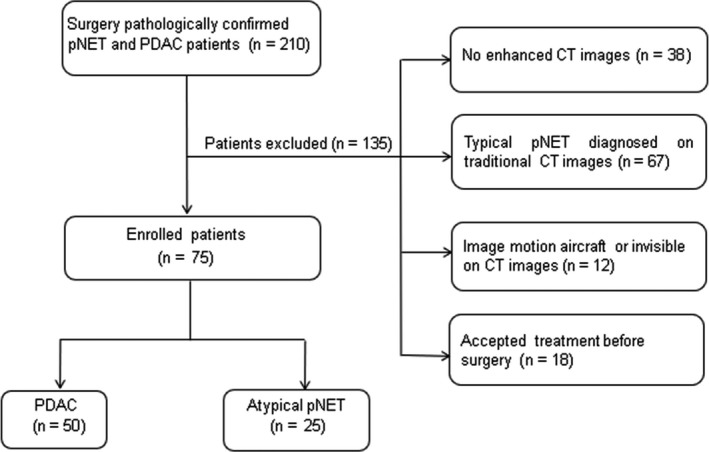
Flowchart of the study population

Finally, 77 pNET patients were preliminarily enrolled in this study population for the following criterion: (a) patients accepted preoperative contrast‐enhanced CT examination; (b) patients with optimal diagnostic images quality; (c) patients who have not undergone therapy before surgery. In addition, we searched pathology database using the search terms “pancreas ductal adenocarcinoma” between May 2015 and May 2017. Then, referencing above inclusion criterion of pNET patients, a total of 50 PDAC patients were selected as control group (Figure 1).

### 2.2 Image acquisition

All patients underwent contrast‐enhanced CT using a 64‐slice MDCT scanner (Discovery CT750 HD, GE Healthcare, USA) in a supine, feet‐first position. Intravenous contrast media 370 mg/mL iopromide (Ultravist 370, Bayer Schering Pharma, Berlin, Germany) was administered at a flow rate of 3.5 mL/s, followed by a 20‐mL saline flush. The total contrast volume was 1.5 mL/kg. Contrast material was injected through the ante‐cubital vein with an 18‐gauge intravenous cannula using a dual‐head injector (Stellant, Medrad, CO, USA), each with an injection time of 20 seconds.

The CT imaging parameters were as follows: automatic tube current; tube voltage, 120 kV; rotation time, 0.5 seconds; detector pitch, 0.984:1; matrix, 512 × 512; table speed, 39.37 mm/rotation; and slice thickness/interval, 0.625 mm. The time of arterial phase scanning was determined by a bolus tracking technique (Smartprep, GE Healthcare Technologies) when a threshold enhancement of 120 HU was achieved in the abdominal aorta. Portal phase imaging was initiated 25‐30 seconds after the completion of arterial phase scanning. All images were transferred to the workstation (AW 4.6, GE Healthcare) for quantitative and qualitative analysis.

### 2.3 Image analysis

All data were evaluated by two independent blinded observers, with more than 5 years of abdominal CT experience. The radiologists were blinded to clinical information and final histological diagnosis but were aware of the age and sex of patients. Any controversy in the image analysis process reached agreement through discussion.

Traditional imaging analysis were achieved by portal phase images with section thicknesses of 2.5 mm on a workstation (AW 4.6, GE Healthcare), including the following information: (a) enhancement pattern of arterial phase(hypervascular or atypical hypovascular); (b) tumor size; (c) tumor margins (well or ill); (d) tumor homogeneity in portal phase (homogeneous or heterogeneous); (e) the presence of pancreatic duct dilatation—a primary pancreatic duct diameter of 3 mm or greater is considered to be dilated.^18^

Texture analysis was achieved on portal phases of CT images using a developed software (Fire Voxel, New York University, New York, USA). DICOM format images were transferred from the picture archiving and communication system (PACS) to Fire Voxel. Radiologists manually draw the region of interest (ROI) along the edge of the lesion on all layers; then, every ROI were automatically fused together to obtain the entire tumor volume information (VOI). For the generated VOI, the software automatically extracted and calculated histological features of the lesion. Then histogram and texture analysis were computed by IBM SPSS 23.0 software (Chicago, IL). The parameters included the following: mean, median, 5th, 10th, 25th, 75th, 90th percentiles, kurtosis, skewness, and entropy.

### 2.4 Pathological evaluation

Histopathological analysis was achieved on postoperative pathology reports by another reader not involved in the CT image analysis, according to the revised 2010 World Health Organization classification criteria.

### 2.5 Statistical analysis

All statistical analyses were completed with IBM SPSS 23.0 (Chicago, IL) and MedCalc (MedCalc Software, Mariakerke, Belgium). *P* value below 0.05 was considered to infer statistical significance.

The measurement consistency between two radiologists was tested by using interclass correlation coefficient (ICC). Normality was assessed using the Shapiro‐Wilk test (*P *≥* *0.05 indicates normal distribution). For differences between PDAC and atypical pNET groups, categorical variables were compared by the *x* ^2^ test and continuous variables were compared by the Student *t* test or Mann‐Whitney *U* test. Applying receiver operating characteristic (ROC) curves analysis to determine the optimal threshold, sensitivity, and specificity of significant parameters for identifying PDACs and atypical pNETs. The comparison between AUC was completed by *Z* test.

## 3 RESULT

### 3.1 Patient and lesion characteristics

**Figure 2:**
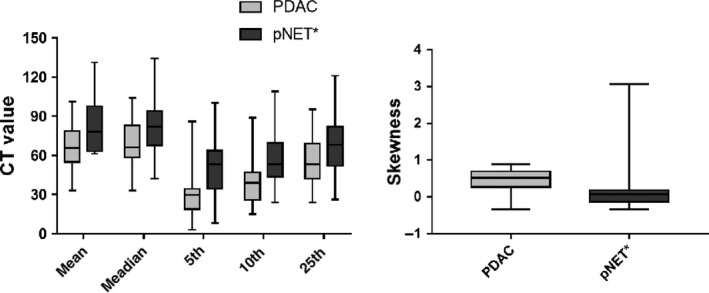
Bar charts of the comparison of CTTA parameters between PDAC and atypical pNET; PDAC, pancreatic ductal adenocarcinomas; pNET, pancreatic neuroendocrine tumors

**Figure 3:**
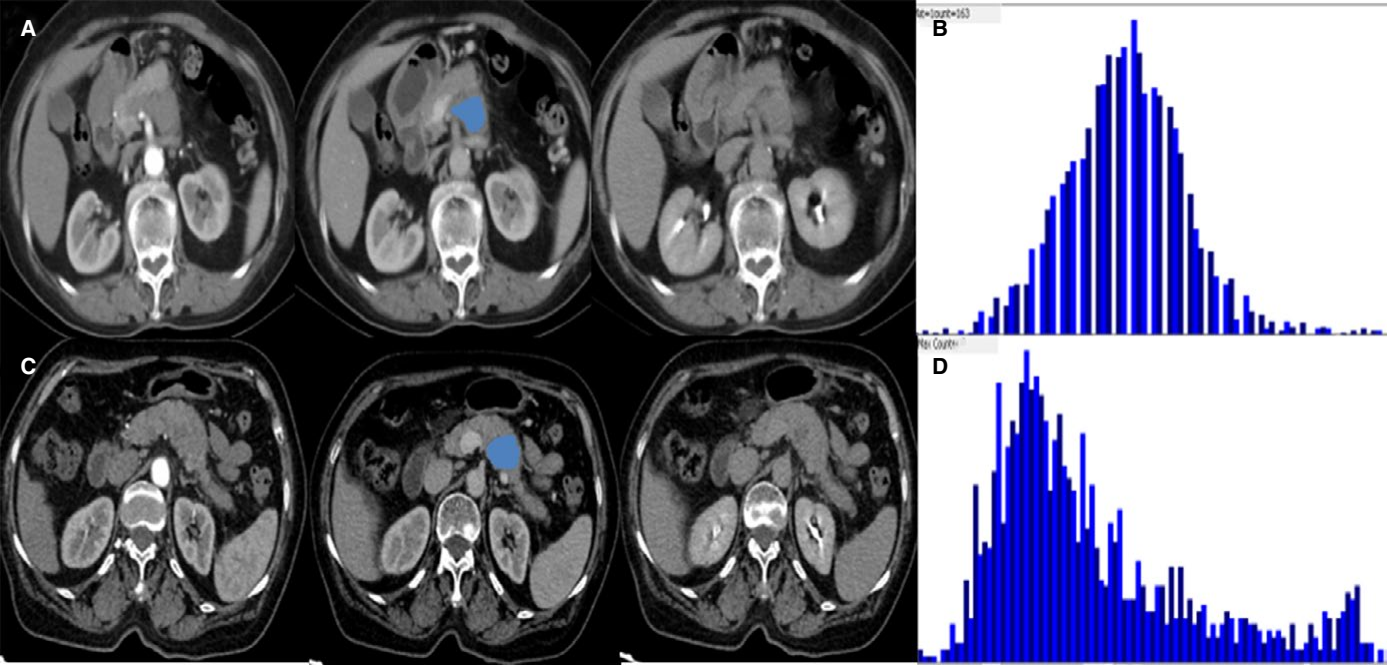
Examples of atypical pancreatic neuroendocrine tumors (pNETs) and pancreatic ductal adenocarcinomas (PDACs) using three‐phase CT scan. A, The arterial phase showed a hyporvascular lesion in pancreatic head. B, The image gray‐level histogram showed a skewness value of 0.31 of pixel‐wise attenuation on portal phase. C, The arterial phase showed a hypovascular lesion in pancreatic body. D, The image gray‐level histogram showed a skewness of 1.1 of pixel‐wise attenuation on portal phase

On the basis of arterial enhancement, 52 pNETs (67%, 52/77) were typical hypervascular and 25 pNETs (32%, 25/77) were atypical hypovascular. Therefore, seventy‐five patients with surgery pathologically (atypical pNET = 25, PDAC = 50) were enrolled in this study finally. Well‐defined margin and homogeneity on portal phase were easier to appear in atypical pNET compared to PDAC (*P *=* *0.032, *P *=* *0.046). However, there was no significant difference in terms of age, sex, tumor size, and main pancreatic duct dilatation between two lesions (*P *=* *0.631, 0.139, 0.064, 0.230, respectively; Table 1). Two sets of typical cases are shown in Figures 2 and 3.

**Table 1:** Characteristics of patients with PDAC and atypical pNET

	PDAC	pNET	*P*
Number of patients	50	25	
Age, y	54.9 (41‐75)	53.8 (45‐68)	0.631
Sex			
Man	31	11	0.139
Woman	19	14	
Tumor size, cm	4.18 (2.3‐11)	3.43 (1.2‐4.7)	0.064
Margin			
Well defined	17	15	**0.032**
Ill defined	33	10	
Main pancreatic duct dilatation			
≥3 mm	35	14	0.230
<3 mm	15	11	
Homogeneity			
Homogeneous	16	14	**0.046**
Heterogeneous	34	11	

Data in parentheses are ranges. Significant differences are in bold.

PDAC, pancreatic ductal adenocarcinomas; pNET, pancreatic neuroendocrine tumors.

### 3.2 Histogram and texture parameters comparison

**Figure 4:**
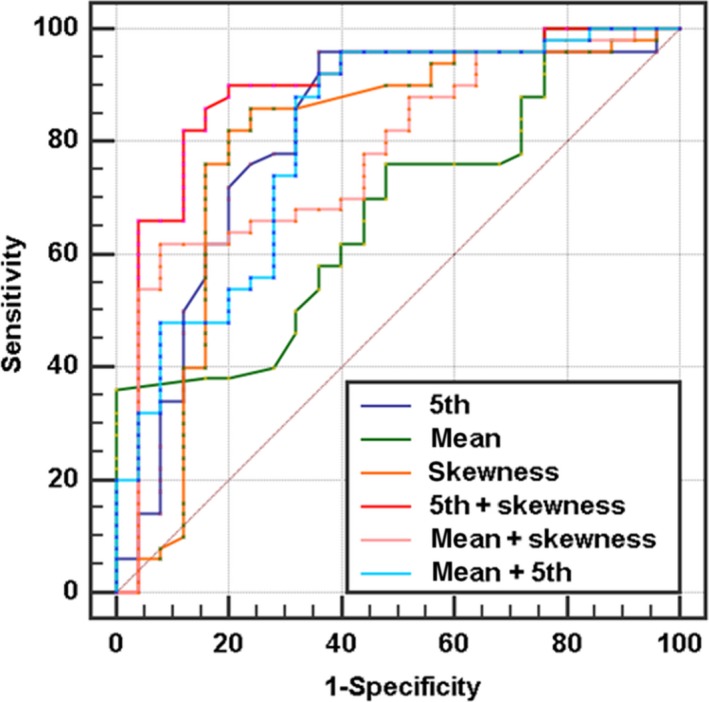
ROC analysis of every significant CTTA parameters. “+” present combined diagnosis. The 5th+skewness has the highest diagnostic efficiency and its AUC is 0.887

The degree of interobserver agreement was excellent (ICC >0.81) for all parameters (Table 2). Therefore, this study chose a measurement value from one radiologist randomly as the final result. The values of mean, median, 5th, 10th, and 25th percentiles were statistically lower in PDAC than atypical pNET (*P *=* *0.006, 0.024, 0.000, 0.001, 0.021, respectively). PDAC had statistically higher skewness than another group (*P *=* *0.017) (Figure 4). However, 75th and 90th percentiles, kurtosis, and entropy did not statistically differentiate these two tumors (*P = *0.232, 0.415, 0.143, 0.291, respectively; Table 3).

**Table 2:** The interobserver agreement between two radiologists of different histogram parameters

Parameter	ICC	95% CI
Mean	0.838	0.744‐0.898
Median	0.848	0.760‐0.904
5th percentile	0.839	0.745‐0.898
10th percentile	0.822	0.718‐0.888
25th percentile	0.810	0.690‐0.880
75th percentile	0.833	0.708‐0.883
90th percentile	0.852	0.765‐0.906
Skewness	0.894	0.831‐0.933
Kurtosis	0.879	0.809‐0.924
Entropy	0.890	0.826‐0.930

ICC, intraclass correlation coefficient; CI, confidence intervals.

**Table 3:** Results of texture analysis of CT value between PDAC and atypical pNET

Parameters	PDAC	pNET	*P* value
Mean^a^	66.36 ± 21.16	80.9 ± 20.64	0.006*
Median^a^	69.5 ± 18.29	81.8 ± 22.84	0.024*
5th percentile^a^	28.51 ± 15.62	50.43 ± 21.88	0.000*
10th percentile^a^	39.26 ± 15.98	57.16 ± 21.51	0.001*
25th percentile^a^	55.39 ± 17.39	66.77 ± 23.17	0.021*
75th percentile^a^	82.42 ± 20.19	88.64 ± 22.77	0.232
90th percentile^a^	95.11 ± 24.03	99.88 ± 23.07	0.415
Skewness	0.45 ± 0.28	0.18 ± 0.67	0.017*
Kurtosis	0.85 ± 0.96	0.61 ± 0.44	0.143
Entropy	2.78 ± 0.41	2.66 ± 0.49	0.291

Data in table are mean ± SD.

PDAC, pancreatic ductal adenocarcinomas; pNET, pancreatic neuroendocrine tumors.

*P <* *0.05.

_a_Units of HU for CT value.

As shown in Table 4, 5th, 10th percentiles, and skewness generated the higher AUC (AUC = 0.811; 0.758; 0.792, respectively) than mean CT value (AUC = 0.678) for differentiating atypical pNET and PDAC. Moreover, mean +skewness and 5th+skewness gained statistically higher AUC values than mean(*P = *0.034, 0.004). Furthermore, 5th+skewness generated the highest AUC (0.887) and corresponding sensitivity and specificity were 90% and 80%, respectively (Table 5).

**Table 4:** Effectiveness of CTTA in discriminating atypical pNET from PDAC

Parameters	AUC	Cut‐off	Sensitivity (%)	Specificity (%)	You‐Index
Mean^a^	0.678 (0.560‐0.781)	60	36	100	0.360
Median^a^	0.675 (0.557‐0.779)	64	52	88	0.400
5th percentile^a^	**0.811 (0.704‐0.892)**	**44**	**96**	**64**	**0.600**
10th percentile^a^	0.758 (0.645‐0.849)	46	74	76	0.500
25th percentile^a^	0.659 (0.541‐0.765)	50	48	84	0.320
75th percentile^a^	0.572 (0.452‐0.686)	88	64	52	0.160
90th percentile^a^	0.547 (0.428‐0.662)	95	58	56	0.140
Skewness	0.792 (0.682‐0.877)	0.15	86	76	0.620
Kurtosis	0.532 (0.413‐0.648)	0.45	60	56	0.160
Entropy	0.572 (0.452‐0.685)	2.81	56	64	0.200
Mean+5th	0.808 (0.701‐0.890)	NS	88	68	0.56
Mean+skewness	0.779 (0.669‐0.867)	NS	62	92	0.54
5th+skewness	**0.887 (0.793‐0.948)**	**NS**	**90**	**80**	**0.700**

Data in parentheses are ranges. “+” present combined diagnosis. Optimal AUC are in bold. ^a^Units of HU for CT value.

PDAC, pancreatic ductal adenocarcinomas; pNET, pancreatic neuroendocrine tumors; AUC, area under the receiver operating characteristic curve.

**Table 5:** Comparison of area under the receiver operating characteristic curve of CTTA parameters

Parameter	5th	Mean	Skewness	Mean +5th	Mean +skewness
Mean	0.134				
Skewness	0.851	0.188			
MEAN +5th	0.914	0.067	0.866		
Mean +skewness	0.733	**0.034**	0.796	0.722	
5th +skewness	0.200	**0.004**	0.129	0.176	**0.044**

Data are *P* values.

*P *<* *0.05 (bold) indicated that the difference was statistically significant.

### 3.3 Pathological result

According to the revised 2010 World Health Organization classification criteria, pNETs and PDAC were categorized. We found in hypervascular pNET group, 52% (27 of 52) were G1 tumors, 31% (16 of 52) were G2 tumors, and 17% (9 of 52) were G3 tumors. In atypical hypovascular pNET group, 24% (6 of 25) were G1 tumors, 40% (10 of 25) were G2 tumors, and 36% (9 of 36) were G3 tumors. In PDAC group, 18% (9 of 50) were well differentiated adenocarcinomas, 48% (24 of 50) were moderately, and 34% (17 of 50) were poorly.

## 4 DISCUSSION

Differentiating quantitatively atypical pNET from PDAC before treatment is difficult but this study had demonstrated an ability to achieve this by CTTA.

In this study, conventional CT features were first analyzed. Similar to previous findings, this study demonstrated homogeneity on portal phase could help differentiate atypical pNET from PDAC. However, for tumor margin and pancreatic duct dilatation, this study had the opposite results compared with Kim JH.^11^ It demonstrated that conventional CT features may have some limitations in the differentiation of atypical pNET and PDAC, which may result in confusion and misdiagnosis. Therefore, CTTA was studied to promote the differential diagnosis.

All voxels in the lesion were arranged from small to large based on CT value, and the corresponding CTTA percentiles were shown in histogram.^19^ For contrast‐enhanced CT, the higher percentiles (75th and 90th) often reflects the blood perfusion in the lesion and the lower percentiles (5th and 10th) often reflects cystic degeneration and necrosis. On the basis of our results, higher percentiles (75th and 90th) did not demonstrate statistically differences between atypical pNET and PDAC, which means that blood variations on portal phase may have limited value. However, lower percentiles (5th, 10th and 25th) of PDAC were significantly lower than those of atypical pNET. This finding may reflect the pathological characteristics of PDAC, frequently relating to cystic degeneration and necrosis because of rapid tumor cell growth.^20^ Thus, the lower percentiles may be important factors in differentiating atypical pNET from PDAC by CT.

Previous studies have indicated that the microenvironment heterogeneity was linked with the non‐uniform distribution of CT attenuation values within tumors.^21^ Skewness can reflect the asymmetry of the histogram and the skewness value can be positive or negative. Positive skew indicates that the tail on the right side of histogram is longer than the left. As the absolute value of skewness increases, the histogram curve deviates farther away from normal distribution. Previous literatures showed that higher grade pNET had statistically higher skewness than lower grade pNET.^22^, ^23^ Similar findings appeared in this study, the skewness of PDAC was greater than atypical pNET, indicating that CT value distribution of former deviated more from Gaussian distribution and majority CT value of PDAC were concentrated on the left of histogram. If validated in larger studies, a higher CT skewness may tend to PDAC instead of atypical pNET and help doctors to select more aggressive surgical approaches including extensive lymph node dissections.

Kurtosis and entropy could, respectively, represent the shape and irregularity of the voxel distribution, which could represent tumor heterogeneity from different perspectives.^24^ Liu SL 14 and Feng *Z* 25reported that kurtosis and entropy based on CTTA could help differentiate benign and malignant tumors. However, we did not find significant difference in kurtosis and entropy between PDAC and atypical pNET. The contradict results may be caused by the following reasons: first of all, the whole tumor was selected for ROI rather than a single axial level to acquire histogram parameters in this study, enabling elimination intralesion heterogeneity; second, the images of this study were from contrast‐enhanced CT rather than unenhanced CT. Although there was no statistically difference in kurtosis and entropy, the values of them gradually increased as tumor malignant degree increased (from pNET to PDAC). This variation trend was in accordance with previous researches.^26^, ^27^ Hence, this study could provide reference value for further large sample research and contribute to the development of CTTA.

This study had several limitations. First, the study population was relatively small, although pNETs are rare tumors. Second, this was a retrospective study with inherent biases in patient selection. Third, only portal phase was used for CTTA. The texture analysis with multi‐phase CT may increase the diagnostic performance. Fourth, this preliminary study used the first‐order parameters of CTTA merely, higher‐order parameters would be included in the next large sample study.

In conclusion, CTTA parameters especially 5th+skewness may contribute to a reduced number of false diagnoses of atypical pNETs as PDACs. It would be useful for surgery planning and the selection of combined treatments.

## CONFLICT OF INTEREST

None declared.
